# Microstructure and Mechanical Properties of Y_4_Zr_3_O_12_-Added Fe–13.5Cr–2W Oxide-Dispersion-Strengthened Steels, Containing High Contents of C and N, Prepared by Mechanical Alloying and Two-Step Spark Plasma Sintering

**DOI:** 10.3390/ma16062433

**Published:** 2023-03-18

**Authors:** Yiheng Wu, Qunying Huang, Ligang Zhang, Yong Jiang, Gaofan Zhu, Jingjie Shen

**Affiliations:** 1 Hefei Institutes of Physical Science, Chinese Academy of Sciences, Hefei 230031, China; 2 University of Science and Technology of China, Hefei 230026, China; 3 School of Materials Science and Engineering, Central South University, Changsha 410083, China; 4 National Institute for Fusion Science, Toki 509-5292, Japan

**Keywords:** oxide-dispersion-strengthened (ODS) steel, mechanical alloying (MA), spark plasma sintering (SPS), microstructure, mechanical property

## Abstract

Oxide-dispersion-strengthened (ODS) steel is considered as a promising candidate structural material for nuclear applications. In this study, the microstructure and mechanical properties of Y_4_Zr_3_O_12_-added Fe–13.5Cr–2W ODS steels, containing high contents of C and N, prepared by mechanical alloying (MA) and two-step spark plasma sintering (SPS), were investigated. The results showed that pure Y_4_Zr_3_O_12_ powders, with a grain size of 3.5 nm, were well prepared with NH_3_·H_2_O addition by the sol-gel method in advance, in order to avoid the formation of some coarse or undesired oxides. W was completely dissolved into the matrix after 48 h of ball milling at 300 rpm, and the main elements were uniformly distributed on the surface of the milled powders. The unexpected face-centered cubic (FCC, γ)/body-centered cubic (BCC, α) dual-phase structure of the sintered specimens, could be explained by the unexpectedly high contents of C and N from the raw powder production process, fast-sintering characteristic of SPS, and inhibitory effect of W on the diffusion of C. The experimental results were approximately consistent with the simulation results from the Thermo Calc software. The temperature combination of 800 °C and 1100 °C during the SPS process, provided a relatively more homogeneous microstructure, while the combination of 750 °C and 1150 °C, provided the highest ultimate tensile strength (UTS), of 1038 MPa, with the highest uniform elongation (UE), of 6.2%. M_23_C_6_, Cr_2_O_3_, M_2_(C,N), and other precipitates, were mainly distributed at grain boundaries, especially at the triple junctions, which led to Cr depletion at grain boundaries.

## 1. Introduction

The structural materials in a fusion reactor will be subjected to high heat loads and neutron fluence [1]. Oxide-dispersion-strengthened (ODS) ferritic steel is considered as one of the potential candidates for the structural material in a fusion reactor, due to its good high-temperature mechanical properties such as tensile and creep strengths [2,3,4], corrosion and oxidation resistance [5,6], and high-dose irradiation tolerance [7,8].

Cr is one of the main alloying elements in ODS steel. Increasing the Cr content will significantly improve the corrosion and oxidation resistance of the steel, while enhancing the solid solution strengthening effect. However, the Cr concentration is required to be less than 16 wt.%, due to aging and irradiation embrittlement issues [9,10]. According to the Fe–Cr binary phase diagram, for steel containing less than 12 wt.% Cr, phase transformation will occur at above 850 °C, and finally isotropic martensite or ferrite can be obtained; while for steel containing more than 13 wt.% Cr, the phase transformation will not occur, and fully ferritic steel can be obtained [11]. However, for Fe–(5, 9, 10, 11, 12, 13, and 13.5) Cr–2W–0.5Ti–0.25Y_2_O_3_ steels, austenite transformations were found in all steels, except for the ODS–13.5Cr steel [12]. This might be due to the presence of minor C and N in other steels, which can act to promote the formation of austenite. Therefore, the Fe–13.5Cr–2W steel could be fully ferritic throughout the sintering process, improve the microstructure stability, and minimize the aging and irradiation embrittlement issues.

Dispersion strengthening by oxide nanoparticles is the core of the strengthening mechanism of ODS steel. The type, size, and distribution state of oxide nanoparticles, and the interfacial relationship between oxide nanoparticles and the matrix, will significantly affect the microstructure and mechanical properties of the steel. Y_2_O_3_ has been widely used in ODS alloys because it is one of the most thermodynamically stable compounds [11,13,14,15,16]. However, the particle size of Y_2_O_3_ is usually large, and thus the strengthening effect is limited. The addition of microalloying elements such as Ti, Zr, Al, Si, Hf, and Ta changes the behavior of Y_2_O_3_, and has an important influence on the composition, size, and distribution of dispersed particles, thus affecting the mechanical properties and irradiation resistance of the alloys [17,18,19,20,21,22,23,24,25,26]. Compared with Ti and Al, Zr is more easily combined with Y_2_O_3_, to form ternary complex oxides, and Y–Zr–O is more stable, because of its high binding energy [27,28,29,30]. Therefore, Zr-containing ODS steels have been extensively studied. However, no matter what microalloying elements are added, the traditional addition method is to mix Y_2_O_3_ and the microalloying elements with other matrix components, to produce oxide nanoparticles. It should be noted, that some coarse or undesired binary oxides such as TiO_2_, ZrO_2_, and Y_2_O_3_ will inevitably remain in the alloy, due to incomplete reactions. For example, Dou et al. found that in Fe–16Cr–0.1Ti–0.35Y_2_O_3_ ODS steel, about 17.7% and 10.1% of the particles were TiO_2_ and Y_2_O_3_, respectively [31]. Y_2_O_3_ was rapidly formed, due to it having the lowest formation energy among Y_2_O_3_, TiO_2_, and Y_2_TiO_5_ [31,32], resulting in the absence of Y in local regions. Moreover, the solid solubilities of Y and O in the ferrite are low [33], while the diffusion coefficient of Y in the ferrite is low as well [34], so TiO_2_ was formed. A study on Fe–15Cr–2W–0.1Ti–4Al–0.63Zr–0.35Y_2_O_3_ steel, also showed that most of the small nanoparticles (< 15 nm) were composed mainly of trigonal δ-phase Y_4_Zr_3_O_12_ particles and a few orthorhombic Y_2_TiO_5_ particles, making up ~97.8% of all oxides in the steel, while no small Y–Al–O or Al–O particles were observed. However, the large particles, which made up ~2% of all oxides, were mainly identified as tetragonal or cubic ZrO_2_, while a few Y–Al–O particles were also found [30]. Pazos et al. [35] found that in Fe–14Cr–2W–0.3Ti–0.3Y steel, most of the analyzed larger particles (> 50 nm) were Y_2_O_3_, while nearly half of the finest nanoparticles were Y–Ti–O particles, with different Y/Ti ratios. Zhang et al. [36] also demonstrated that the smaller particles tended to be Y_4_Zr_3_O_12_, whereas the larger particles generally existed as Y_2_O_3_.

In fact, some studies have been conducted to solve this problem. The main idea was to combine Y with other microalloying elements in advance, to ensure their sufficient contact and combination. Then, the as-received Y–M–O (M = microalloying element) powders would react with other matrix components. Simondon et al. [37] synthetized pyrochlore-type Y_2_Ti_2_O_7_, by mixing Y_2_O_3_ and TiO_2_ by mechanical milling. Liu et al. [38] prepared Y_2_Ti_2_O_7_ nanopowders by the combination of hydrogen plasma–metal reaction and annealing, and explained that Y_2_Ti_2_O_7_ was transformed from the amorphous state during mechanical alloying (MA), to crystalline precipitates during hot isostatic pressing (HIP). In addition, Wu et al. [39] produced Y_4_Zr_3_O_12_ nanopowders by the stearic acid sol-gel method, and directly mixed them with other elemental powders by MA. Similarly, Sun et al. [40] prepared Fe–1.5Y_2_O_3_ powders, with sol-gel and hydrogen reduction methods, in advance, to promote more uniform distribution of oxide particles in the matrix. Overall, this preparation idea is worth studying and can be further improved.

MA is the main and effective manufacturing process for ODS steel [41,42]. The particles in the ball milling container are subjected to a long period of intense impact, collision, friction, and shearing action by the milling container and milling balls, thus the particles will undergo severe plastic deformation and repeated cold welding and fracture [42]. In this process, the alloying elements are solidly dissolved into the matrix, resulting in supersaturated, solid solution, alloyed powder. In fact, all atomized or elemental powders are mechanically alloyed with Y_2_O_3_ powders, to obtain a homogeneous dispersion of nano-oxides in the matrix [43]. Then, the alloyed powders will be consolidated under high temperature and pressure. Spark plasma sintering (SPS) is a consolidation method, employing pulsed direct current and uniaxial pressure to achieve rapid sintering [44]. The high heating and cooling rates of the SPS method lead to a dense structure, with lower grain growth [45]. Compared with the traditional consolidation techniques such as HIP and hot extrusion (HE), SPS shows some unique advantages, including a low sintering temperature, short processing time, convenient operation, and the ability to control any stage of the sintering process [46]. The SPS technique has been widely applied to prepare ODS alloys [47,48,49,50]. The two-step sintering method has been widely performed in ceramics, to improve the densification of materials [51], and Mihalache et al. [52] applied the two-step sintering method to ODS ferritic steels to improve the relative density. However, there was no clear basis for the design of the lower temperature platform.

The aim of this study is to assess the potential of MA and two-step SPS, to fabricate Y_4_Zr_3_O_12_-added ODS ferritic steels. The composition of the ODS steels would be designed first, especially with regard to the Cr content. The Y_4_Zr_3_O_12_ powders were prepared by the sol-gel method in advance, to avoid the formation of ZrO_2_ and Y_2_O_3_, and part of the preparation process from [39] was followed. However, differently to [39], NH_3_·H_2_O was used in this study, to promote the more stable binding of Y^3+^ and Zr^4+^ in the solution, and the effect of different calcination temperatures on the evolution of Y_4_Zr_3_O_12_ was further investigated. Then, the powders prepared by MA under different ball-milling processes, and the steels consolidated by two-step SPS under different sintering conditions, were investigated carefully. Unexpectedly, the sintered steels had a face-centered cubic (FCC, γ)/body-centered cubic (BCC, α) dual-phase structure, and the abnormal phase transformation is especially discussed. This study will have a certain significance for the design and preparation of structural materials for fusion reactor applications and can also provide a reference for the strengthening routes of new materials.

## 2. Materials and Methods

### 2.1. Materials and Preparation

**Figure 1:**
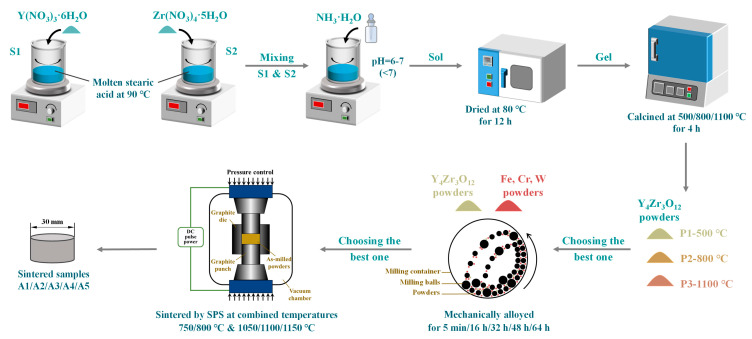
Schematic diagram of the preparation process.

**Figure 2:**
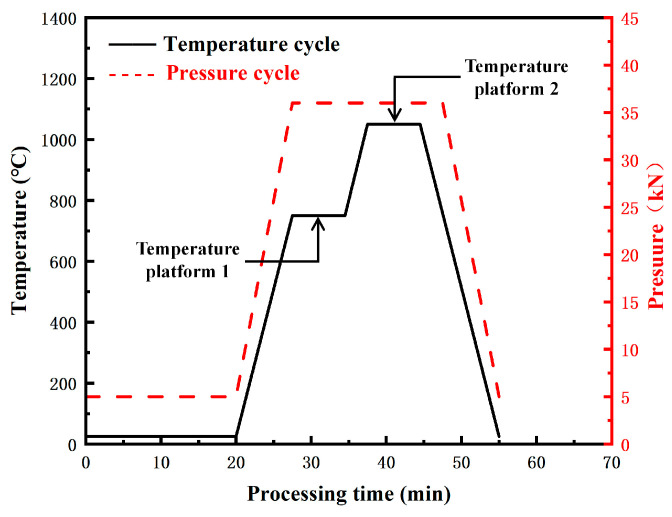
Illustration of the applied temperature and pressure sintering profile.

A schematic diagram of the preparation process employed in this study is presented in Figure 1. The Y_4_Zr_3_O_12_ nanopowders were produced by the sol-gel method [39]. The Y(NO_3_)_3_·6H_2_O (purity 99.5%) and Zr(NO_3_)_4_·5H_2_O (purity > 98%) raw powders, with proper ratios, were added to adequate molten stearic acid at 90 °C. The as-received two solutions were named S1 and S2, respectively. S1 and S2 were mixed together after being stirred separately for 0.5 h. NH_3_·H_2_O was added into the mixture to promote the binding of Y^3+^ and Zr^4+^, and the pH was adjusted to 6–7 (< 7), then the sol was obtained. The sol was dried at 80 °C for 12 h, to yield gels. The gels were respectively calcined at 500 °C, 800 °C, and 1100 °C, for 4 h, to produce Y_4_Zr_3_O_12_ powders, and the powders were named as P1, P2, and P3, respectively. The 0.6 wt.% as-produced Y_4_Zr_3_O_12_ powders, obtained at a proper temperature, were mechanically alloyed with Fe, Cr, and W elemental powders with compositions of Fe–13.5Cr–2W, using a high-energy planetary ball mill, in a high-purity argon atmosphere, with a ball-to-powder mass ratio (BPR) of 15:1, for 0–64 h, at 300 rpm. According to Diouf and Molinari [53], the grain size of the sintered alloy kept memory of the size of the initial powders, hence very fine powders were used in this study to improve the fine-grain-strengthening effect. The chemical compositions of the alloyed powders, milled for a proper time, and the sizes of the raw powders, are listed in Table 1. Y_4_Zr_3_O_12_ was not the only source of oxygen in the powders. The oxygen on the surface of the raw powders and in the atmosphere was readily absorbed into the activated powders during, and after, the ball milling process. The effect of the unexpected high contents of C and N on the microstructure and mechanical properties, will be discussed in Section 3.1.3 and Section 3.2. The alloyed powders were consolidated in a graphite die and graphite punches by SPS (FCT group, Frankenblick, Germany). Graphite paper was used inside the mold chamber, to prevent adhesion between sample and mold during the sintering process. The SPS compacts were heated with a heating rate of 100 °C/min, up to the temperature platform 1, which was chosen as 750 °C or 800 °C, then the samples were heated with the same heating rate up to the temperature platform 2 which was chosen as 1050 °C, 1100 °C or 1150 °C, respectively. The dwelling time at both temperature platforms was 7 min (Figure 2). An axial pressure of 50 MPa was applied throughout the heating stage. The compacts were then cooled down to room temperature (RT), with a cooling rate of approximately 100 °C/min. Finally, the as-received samples, with a diameter of 30 mm, were ground by a grinding wheel, to remove the graphite and carburized layers on the surface of the samples. In this study, the “sample” represents a sintered steel column, while the “specimen” represents a test piece obtained from the sintered steel column.

### 2.2. Characterization Methods

X-ray diffraction (XRD) was carried out on the powders and the sintered specimens, to analyze the crystal structure, using an X-ray diffractometer with a Cu Kα target (X’ Pert, PANalytical, Almelo, The Netherlands). The size distribution of the as-milled powders was measured with a laser diffraction particle size analyzer (Mastersizer 3000, Malvern Instruments, Malvern, UK). The microstructure of the powders and ODS steels was observed with a field emission scanning electron microscope (FE-SEM; ΣIGMA, ZEISS, Oberkochen, Germany), equipped with an X-ray energy dispersive spectrometer (EDS) and an electron backscatter diffraction (EBSD) detector. The element distribution and composition of specimens were detected with an electron probe micro-analyzer (EPMA; SHIMADZU EPMA-8050G, Kyoto, Japan). The microstructure of the nanopowders was characterized using a transmission electron microscope (TEM; JEM 2100F, JEOL, Akishima, Japan) and high resolution transmission electron microscope (HRTEM). The contents of carbon, nitrogen, and oxygen were determined with a carbon–sulfur analyzer (Eltra CS2000, Haan, Germany) and oxygen–nitrogen analyzer (Eltra ON900, Haan, Germany). The content of Cr was determined by a manual titration method (NACIS/C H116:2020), and the contents of other compositions were detected with an inductively coupled plasma optical emission spectrometer (ICP-OES; Thermo ICP6300, Thermo Fisher Scientific, Waltham, USA). The Thermo Calc software, with TCFE12 database, was used to obtain the equilibrium phase compositions at different temperatures, for the steels with different compositions.

### 2.3. Property Tests

The densities of the consolidated specimens were measured by the Archimedes method, using the following equation:

$$\rho =\frac{\rho_{L}\times m_{1}}{m_{1}-m_{2}}$$

where *ρ* is the density of the specimen, *ρ_L_* is the density of ultrapure water, *m*_1_ is the weight of the specimen in air, and *m*_2_ is the weight of the specimen in ultrapure water.

**Figure 3:**
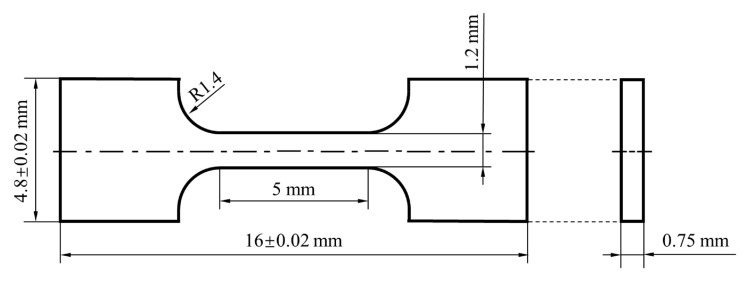
Shape and dimensions of the miniature tensile specimen.

Vickers microhardness measurements were performed at RT, under a load of 200 g and dwelling time of 15 s. The measurements were repeated nine times in total for each specimen, and the nine indentations were distributed as 3 × 3, in a square area. Tensile tests were conducted for miniature specimens (Figure 3), with a displacement rate of 0.3 mm/min, at RT, which were repeated three times for each condition to verify the reliability of the results. The morphology of the fracture surfaces of the tensile specimens was observed with SEM.

## 3. Results and Discussion

### 3.1. Microstructure Characterization

#### 3.1.1. Characterization of Y_4_Zr_3_O_12_ Powders

**Figure 4:**
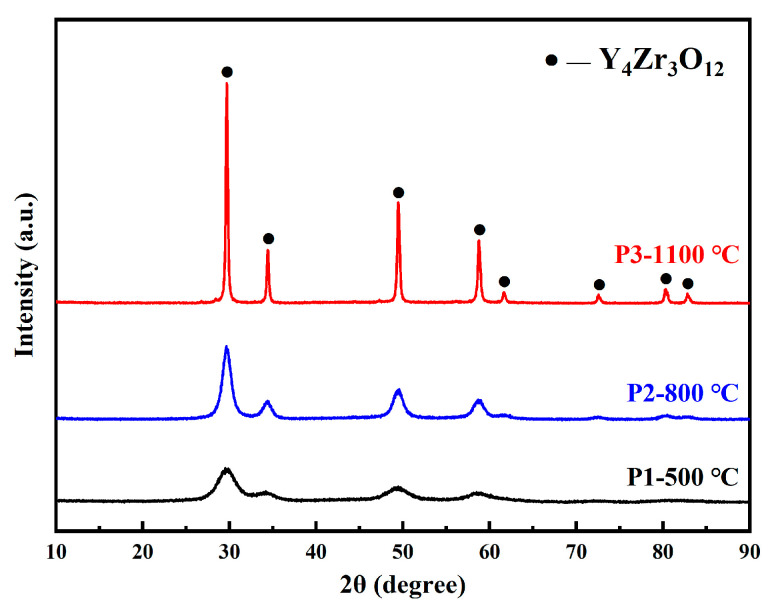
XRD patterns of the Y_4_Zr_3_O_12_ powders calcined at different temperatures.

**Figure 5:**
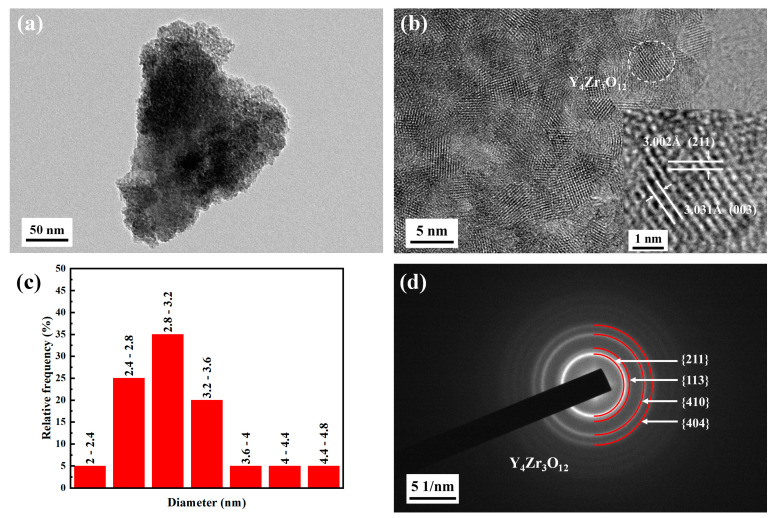
(**a**) TEM image, (**b**) HRTEM image, (**c**) Particle size distribution, and (**d**) SAED image of the Y_4_Zr_3_O_12_ powders calcined at 500 °C for 4 h (specimen P1).

Figure 4 shows the XRD spectra patterns of the Y_4_Zr_3_O_12_ powders calcined at different temperatures. The diffraction peak intensity of specimen P1 was lower, which indicates a low crystallinity of the powders, due to being formed at low temperature. A broadening phenomenon of diffraction peaks was also observed in specimen P1, indicating that the grains were fine or that internal stress existed in specimen P1. The crystallinity increased with the increase in calcination temperature. The grain sizes of specimens P1, P2, and P3 were 3.5 nm, 7.2 nm, and 54.3 nm, respectively, which were calculated by the Debye–Scherrer formula. All the diffraction peaks seemed to correspond to Y_4_Zr_3_O_12_ with a rhombohedral structure (JCPDS: 29-1389, Space group: R-3m (148)). However, the diffraction peaks also nearly corresponded to other substances, with a composition of Y–Zr–O, thus the phase structure required further identification. Considering that a large grain size of nanopowders would deteriorate the properties of the steel, and the grains would grow further during the SPS process, hence the P1 powders were chosen as the raw material to be mechanically alloyed with other powders. Figure 5a shows the TEM image and morphology of specimen P1, and nearly spherical powders, with size below ~6 nm, were obtained. Figure 5b displays an HRTEM image of specimen P1, the internal stress could not be completely released due to the low calcination temperature, hence there was a certain degree of lattice distortion in the powders. The HRTEM image of a typical grain is also shown, the interplanar distances of 3.002 Å and 3.031 Å are, respectively, consistent with the (211) and (003) planes of Y_4_Zr_3_O_12_. Figure 5c shows the grain size distribution statistical chart obtained from Figure 5b, and the average grain size was measured to be 3.1 nm, which was consistent with the result from XRD. The grain size results showed that the addition of NH_3_·H_2_O indeed contributed to the further refinement of the grains of Y_4_Zr_3_O_12_ powders [39]. Moreover, Figure 5d shows a selected area electron diffraction (SAED) pattern of specimen P1. The four diffraction rings correspond to {211}, {113}, {410}, and {404} families of crystal planes of Y_4_Zr_3_O_12_. All the above results proved that the pure Y_4_Zr_3_O_12_ powders were well prepared. The as-received Y_4_Zr_3_O_12_ powders were mixed with other elemental powders to prepare alloyed powders, by MA.

#### 3.1.2. Influence of Milling Time on Microstructure of the MA Powders

The properties of MA powders strongly depend on the control of the MA process, including rotation speed, milling time, BPR, large/small milling ball mass ratio, process control agent (PCA), milling atmosphere, and temperature of milling [42]. Considering that the powders used in this study were very fine, the BPR was chosen as 15:1 and the large/small milling ball mass ratio was chosen as 1:5. As the dissolution of W was regarded as a key signal of effective ball milling [54], the rotation speed was chosen as 300 rpm, to promote the dissolution of elemental W [55]. To avoid contamination from PCA and oxygen, PCA was not added, and the milling process was carried out in a high-purity argon atmosphere. In addition, the intermittent milling method was applied, to avoid overheating the powders. The MA powders were ball milled for 5 min, 16 h, 32 h, 48 h, and 64 h, to study the effect of milling time on them.

**Figure 6:**
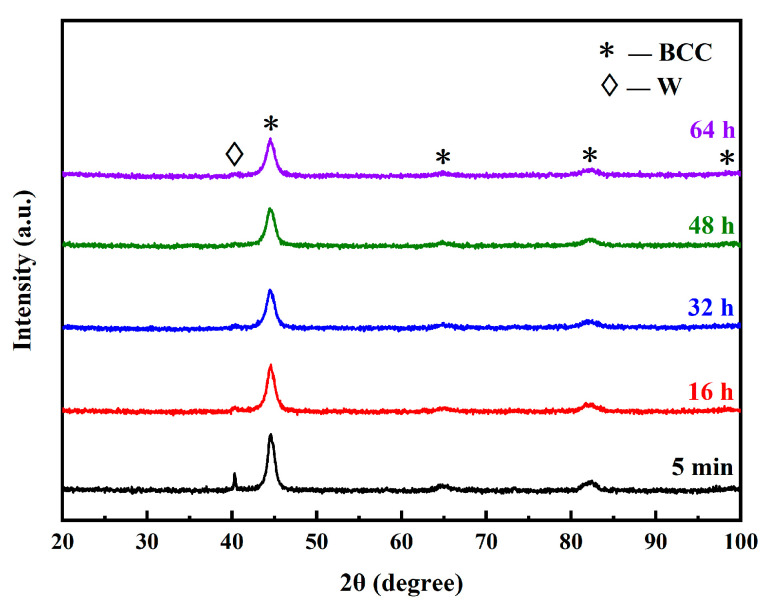
XRD patterns of the alloyed powders with different milling times.

Figure 6 shows the XRD patterns of the alloyed powders with different milling times. With the increase in milling time, the (Fe, Cr)_(110)_ peak width increased, due to the grain refinement and internal stress, while the W peak intensity decreased. When the milling time extended to 48 h, the W peak disappeared, which means that W was completely dissolved into the matrix. Other substances (such as Y_4_Zr_3_O_12_) were undetectable by XRD, due to their low contents.

**Figure 7:**
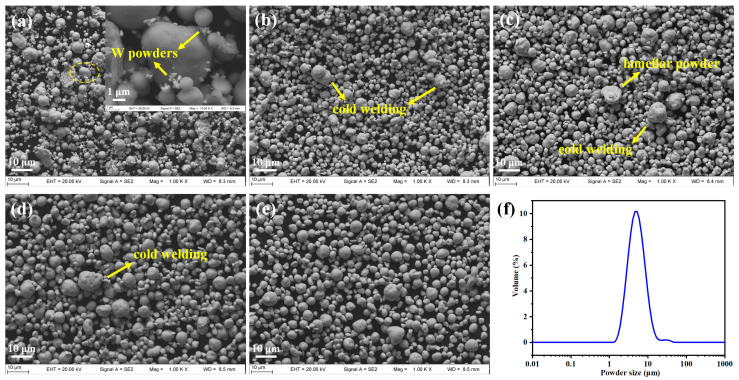
SEM images of powders after different milling times: (**a**) 5 min, (**b**) 16 h, (**c**) 32 h, (**d**) 48 h, and (**e**) 64 h; and (**f**) Distribution of powder size after 48 h of ball milling.

Figure 7 mainly presents the morphology of the powders after different milling times, investigated with SEM. W powders were attached around (Fe, Cr) powders at the initial stage of ball milling, while many powders still maintained the initial spherical shape. A small amount of the particles were deformed, and coldly welded, under the impact of the milling balls and milling container (Figure 7a). It is well known that particles are repeatedly plastically deformed, coldly welded, and fractured during MA, and finally reach a dynamic stable state [42,47,55]. As the ball-milling time increased to 16 h, almost all the particles were irregularly shaped (Figure 7b), and the particles tended to be welded together to form large particles. Thus, the average particle size increased. The lamellar powder morphology caused by plastic deformation was observed after 32 h of milling (Figure 7c). When the milling time extended to 32–48 h, the effect of cold welding between particles continued to increase, and it could be clearly seen that several small particles made up a large particle (Figure 7c,d). After that, the particles were crushed by a violent collision of grinding balls and the average particle size decreased (Figure 7d,e). It could be estimated that the coarsening caused by welding would be balanced by the refining caused by crushing, and the particle size would tend to be stable after a long period of ball milling.

**Figure 8:**
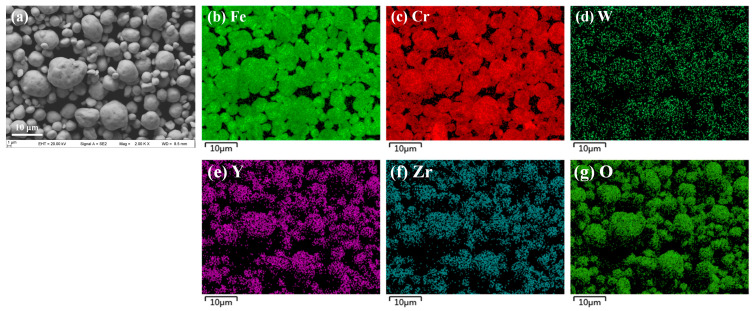
**Morphology and corresponding EDS mapping results of** the powders after 48 h of ball milling: (**a**) Morphology, (**b**–**g**) EDS mapping results.

On balance, the ball-milling time was chosen as 48 h, to avoid potential contamination from an excessive ball-milling time [54], and the rotation speed of 300 rpm was sufficient to facilitate the complete dissolution of W. The powder size distribution, counted by a laser diffraction particle size analyzer, after 48 h of ball milling, is shown in Figure 7f. The value of D_50_ of the alloyed powders after 48 h of ball milling, was only 5.33 μm, with the distribution mainly ranging from 3 μm to 8 μm, which indicates that the alloyed powders used in this study were extremely fine [47,55,56,57]. The element distributions in the powders, observed with SEM-EDS after 48 h of ball milling, are shown in Figure 8. These revealed that the elements were evenly distributed on the surface of the powders. In conclusion, milling for 48 h was required to obtain refined powders with a homogeneous element distribution, which was favorable for the SPS process.

#### 3.1.3. Influence of SPS Temperature Combination on Microstructure of the Sintered Samples

The alloyed powders, after 48 h of ball milling, were consolidated by SPS, and the temperature and pressure cycles are schematically illustrated in Figure 2. Based on the obvious displacement of the punch at about 750 °C, the two temperature platforms were adopted, to complete the sintering process. The specific sintering conditions and relative densities of the specimens are listed in Table 2. The two temperature platforms, together affected the relative densities of the sintered specimens, which was not only determined by the maximum temperature. At the temperature of platform 1, the powders became relatively soft and sintering necks tended to be formed, while the higher temperature at platform 1 helped to facilitate the short-distance movement of the particles, and to ensure a better fit between particles and particles. Considering that a temperature of 1150 °C would cause the samples to melt, sample A4, with the highest relative density, was considered as a “relatively better sample” and was further studied in some respects.

**Figure 9:**
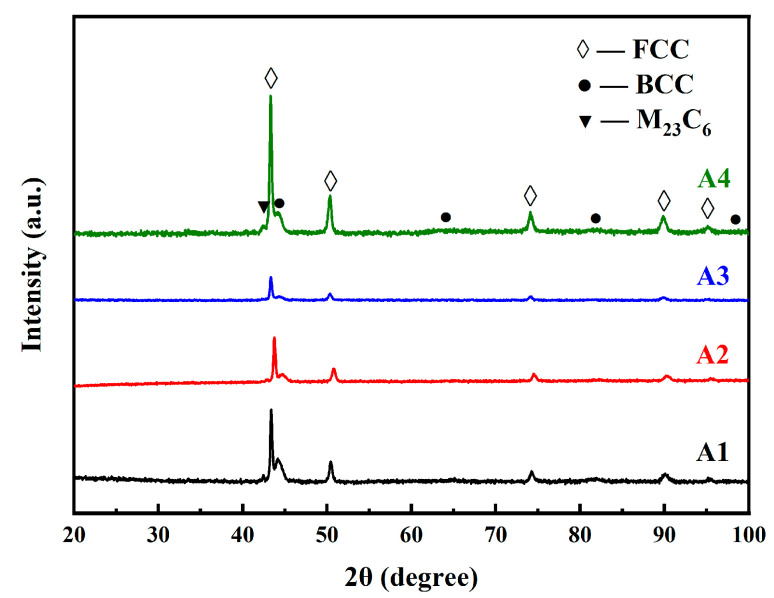
XRD patterns of the sintered specimens.

The XRD patterns of specimens A1–A4 are shown in Figure 9. They indicate that the sintered specimens were all α-γ dual-phase, while γ was the main phase. However, according to the Fe–Cr phase diagram [11], Fe–13.5Cr should be fully ferritic throughout the heating process, and W should further shrink the austenitic phase zone. Thus, Fe–13.5Cr–2W would not undergo austenite transformation at high temperature [12]. The contents of some common austenitizing elements, of the as-milled powders, are listed in Table 1. The results show that the contents of C and N were much higher than expected, which was the origin of the austenite transformation. There were also some M_23_C_6_ precipitates in the specimens. Furthermore, the contents of C and N in the raw powders and the as-sintered steel were detected, and the results are displayed in Table 3. This shows that the C and N were almost exclusively derived from the raw Fe powders prepared by atomization, which were claimed to be qualified. In addition, no new C or N contamination was added into the steels during the sintering process. To eliminate the interference of accidental factors, a new batch of Fe powders, with a particle size of 5 μm, produced by another company, using an electrolytic method, were prepared, and the contents of C and N in the two kinds of Fe powders are shown in Table 4. The contents of C and N are seen to be similar between the two kinds of Fe powders, which was unexpected, and seemed to be close to the maximum naturally dissolved amounts of C and N. A possible explanation for this result, is the different relative contents of C and N in raw Fe powders with different sizes, during the Fe powder production process. It is generally recognized that powders with a wide size distribution are prepared simultaneously during the production process, and then particles of various sizes are sieved. In general, although companies should check the composition of the powders, which might be tested qualified, they might not check the compositions of powders with different sizes. The activity of the very fine Fe powders tended to be higher than the coarse powders, and the specific surface area tended to be larger as well. Therefore, the relative contents of C and N of fine Fe powders, were higher than those of coarser Fe powders. It is important to note that powder production companies should be concerned about this potential engineering issue.

**Figure 10:**
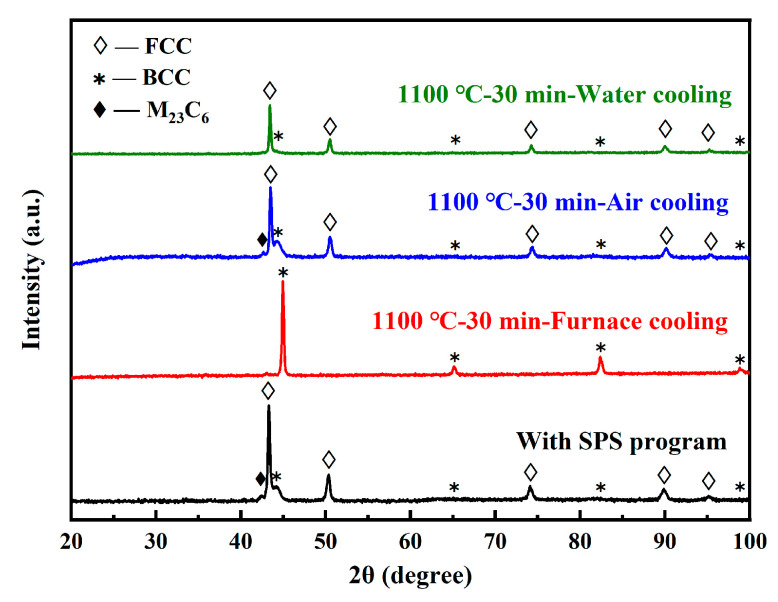
XRD patterns of the sintered specimens before and after different heat treatments.

**Figure 11:**
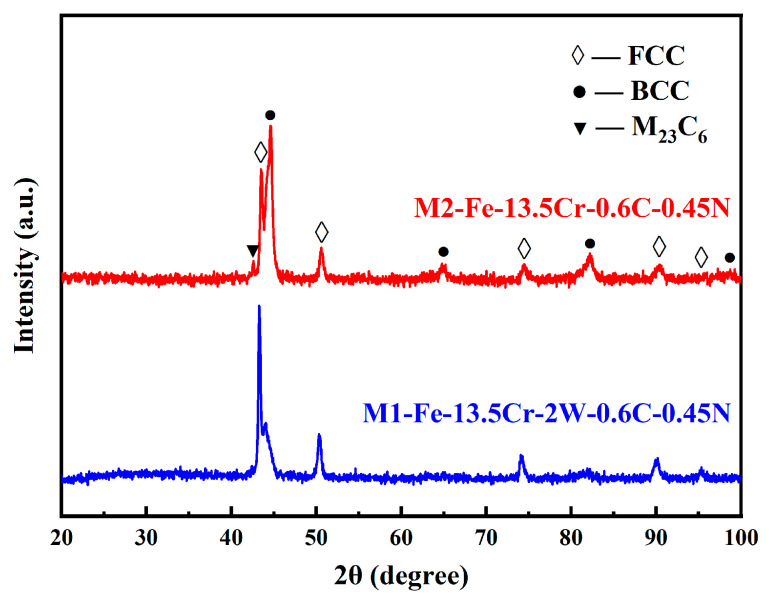
XRD patterns of specimens M1 and M2.

However, the appearance of austenite at RT, found in the Ni-free and Mn-free steels, still seemed somewhat unusual. To further explain the dual-phase of the sintered specimens at RT, the following two exploratory experiments were carried out, including the effects of the cooling rate and the absence of Y_4_Zr_3_O_12_ or W. Firstly, in order to simulate the maximum temperature during the sintering process, the specimens were heated to 1100 °C and held for 30 min, in air, and then cooled down to RT with different cooling rates. The cooling conditions are listed in Table 5. The oxide layers on the surface of the specimens after heat treatment were ground with SiC abrasive paper and then polished for XRD analysis, and the results are shown in Figure 10. Based on the cooling rate, the specimens were ranked as H2 < H1 < H3 < H4. Only specimen H2, with the lowest cooling rate, was fully ferritic at RT, while the other three specimens were all α-γ dual-phase. This revealed that the phase structure was related to the cooling rate. On the other hand, the effect of some elements on austenite stability also needed to be further investigated. Fe–13.5Cr–2W–0.6C–0.45N (named as M1, without Y_4_Zr_3_O_12_) and Fe–13.5Cr–0.6C–0.45N (named as M2, without Y_4_Zr_3_O_12_ and W), were prepared with the same MA and SPS conditions as sample A4, to study the effect of Y_4_Zr_3_O_12_ and W on austenite stability, during the cooling stage. The XRD patterns of specimens M1 and M2 are shown in Figure 11. Combined with the results in Figure 9, it can be concluded that the phase structure at RT was hardly affected by the absence of Y_4_Zr_3_O_12_. However, the proportion of α-Fe became significantly higher with the absence of W, which demonstrates the stabilizing effect of W on the austenite. The mechanism of W’s effect on the phase transformation in Ni-free and Mn-free Fe–Cr steels containing high contents of C and N, is not clear yet.

The stabilizing effect of W on austenite was surprising, since W is a ferrite stabilizer, thermodynamically. Similarly, Nb is also known to be a ferrite stabilizer in thermodynamics, and will raise the A_e3_ temperature [58]. However, kinetically, the addition of a small amount of Nb will greatly delay the γ→α phase transformation. There is a large misfit between the Nb atoms and Fe lattice [59,60], thus Nb tends to segregate to the grain boundaries and reduce the energy of grain boundaries. There is also a drag effect of Nb on phase interface migration in the phase interfaces. In addition, the strong interaction between Nb and C will inhibit the diffusion of C. The inhibitory effect of Nb on γ→α transformation is mainly attributed to the solute drag effect [58,61,62]. The effect of W on γ→α transformation is rarely analyzed from the perspective of kinetics. There is a strong interaction between W and C as well. The explanation of austenite stabilization will be further studied from the perspective of kinetics in the future. The phase transformation during the process of SPS can be described as follows. (i) Heating stage: the ferrite transformed to austenite gradually, and there was no ferrite or a fraction of untransformed ferrite at the highest temperature [63,64]. (ii) Cooling stage: the austenite at high temperature began to change back to ferrite. However, when the cooling rate was fast enough, there was not enough time for austenite to transform completely, resulting in its partial retention at RT. Pure ferrite could be obtained with a sufficiently low cooling rate. In other words, the sintered samples should be completely ferritic at RT, the unexpected contamination of C and N made these samples become dual-phase. In the meantime, the excessive cooling rate and the presence of W, also contributed to the results described above.

**Figure 12:**
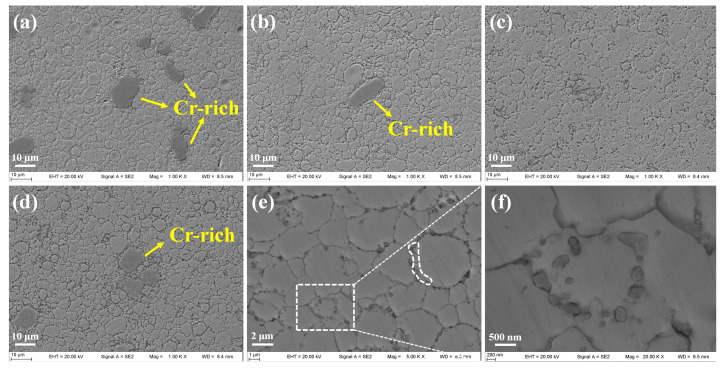
SEM images of sintered specimens: (**a**) A1, (**b**) A2, (**c**) A3, and (**d**) A4; (**e**) and (**f**) Precipitates of A4.

**Figure 13:**
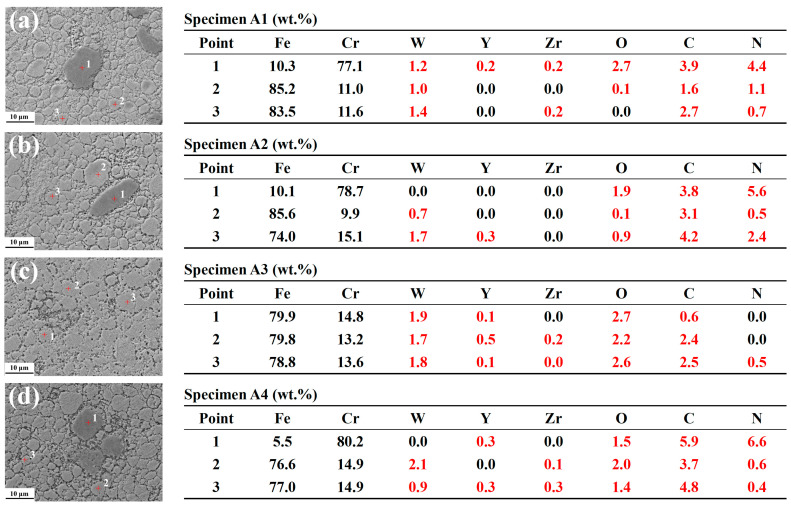
EDS results of sintered specimens: (**a**) A1, (**b**) A2, (**c**) A3, and (**d**) A4 (red results represent the inaccuracy).

Microstructural observations indicated a significant influence of the sintering temperature combinations on the microstructure of the steels. Figure 12 shows SEM observation micrographs of the sintered specimens. Considering the particle size of the very fine as-milled powders, and the as-milled powders’ morphology, shown in Figure 7, the as-sintered specimens preserved some morphological and dimensional features of the alloyed powders, to a certain extent. Figure 12a shows several large and dark areas, which are identified as Cr-rich phases in Figure 13a. A relatively small number of Cr-rich phases can also be seen in Figure 12b,d, while the Cr-rich phase is barely visible in Figure 12c, due to the higher temperature at platform 2. During the SPS process, the following mechanisms began to operate: surface activation, powder and element diffusions, surface melting, the formation of necks between powders, and plastic flow, which combined to influence the microstructural evolution in the sintered alloys [65,66]. Figure 12e,f show the precipitates of specimen A4, distributed both at grain boundaries and within grains. A high number density of nano-scale particles, ranging from ~20 nm to ~400 nm, were arranged in circles and chains, which are marked by a white rectangle and an irregular closed curve, respectively, indicating that they interacted with the extended defects, such as grain boundaries and dislocations. The EDS point scanning mode was used to measure the elemental content of the matrix, and the results are shown in Figure 13. The element compositions of specimens A3 and A4 were closer to the nominal compositions, and more stable due to the higher sintering temperatures, which facilitated the “flow” of the elements. Since specimen A1 contained more Cr-rich phases, the Cr content in the matrix was lower than expected. The distribution of Cr in specimen A2 was not quite even, due to the lower temperature at platform 1. As the measurement of light elements with EDS is not accurate, EPMA, equipped with a wavelength dispersive spectrometer, was adopted for further analysis.

**Figure 14:**
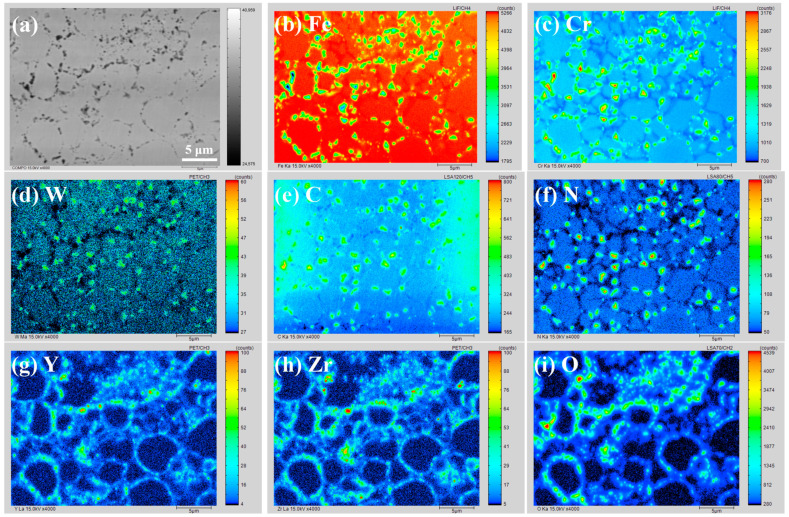
Morphology and corresponding EPMA mapping results of a typical area in specimen A4: (**a**) Morphology, (**b**–**i**) EPMA mapping results.

Taking specimen A4 as an example, a typical area was chosen for EPMA mapping analysis, and the results are displayed in Figure 14. The distribution characteristics of the elements within the matrix and at grain boundaries, were different. As specimen A4 was well prepared by SPS, all the elements were evenly distributed within the matrix, except for C. C was evenly distributed regionally within the matrix where ferritic and austenitic phases were both present, while the solubility of C in the ferrite and austenite was different. The evenly distributed W, also impeded the diffusion of C in the Fe matrix. Cr was continuously distributed at grain boundaries. Cr was particularly locally enriched at the triple junctions, which suggests the formation of Cr-rich precipitates. The Cr-rich precipitates would result in Cr depletion at grain boundaries, which would decrease the stability of the grain boundaries. The Cr-rich regions correspond to the dark “hole-like” areas in the morphology image (Figure 14a). Compared with the ordinary grain boundaries, new phase nucleation and void creation are more likely to occur at triple junctions; and triple junctions can be favorable channels for the diffusion of solute atoms, due to the potential for larger space, a looser structure, more severe stress concentration, a more chaotic atomic arrangement, and more vacancies, dislocations, and other defects [67,68,69,70,71]. As a result, Cr-rich precipitates, and other small-scale compounds, were more likely to precipitate at triple junctions. Moreover, many precipitates were also present at the ordinary grain boundaries. The high dislocation density at grain boundaries provided energy for the nucleation of precipitates, while the high grain boundary density, resulting from the fine grains, also provided more sites for the nucleation of precipitates. W was slightly enriched at some of the triple junctions as well. The distribution of C at the triple junctions was partly overlapped with that of Cr and W, which indicated the formation of carbides such as M_23_C_6_ (M = Fe, Cr, W), M_7_C_3_ (M = Fe, Cr, W), and WC. There was also a certain enrichment behavior of N at the triple junctions, which correlated with that of C, implying that compounds containing C and N might have formed. 

Y, Zr, and O were highly coincident, indicating that Y_4_Zr_3_O_12_ particles were stable in composition during the preparation process. Liu et al. demonstrated that pre-prepared Y_2_Ti_2_O_7_ powders became amorphous during the MA process, while the powders remained stable in composition and did not dissolve in the Fe matrix [38]. Y_4_Zr_3_O_12_ with a higher binding energy than Y_2_Ti_2_O_7_ would also remain stable in composition and not dissolve in the Fe matrix during the MA process. A typical Y_4_Zr_3_O_12_ particle, with a diameter of 8 nm, was observed in Fe–15Cr–2W–0.35Ti–0.6Y_4_Zr_3_O_12_ steel, which was prepared by a similar sol-gel method, as used in this study, MA, and HIP [39]. As a result, Y_4_Zr_3_O_12_ would remain stable during the SPS process. The Y_4_Zr_3_O_12_ particles were continuously distributed at the grain boundaries and dispersed within the matrix, which showed that there were many Y_4_Zr_3_O_12_ particles distributed in the steels. Besides, the distributions of Cr and O were also overlapped in some areas, which revealed the formation of some Cr_2_O_3_ particles. The MA process was beneficial to the uniform distribution of carbides, nitrides, and carbonitrides. However, the oxides were enriched in some areas, where defects were regionally distributed. The high-density defects impeded the diffusion of elemental O, and promoted the combination of O with oxyphilic elements.

**Figure 15:**
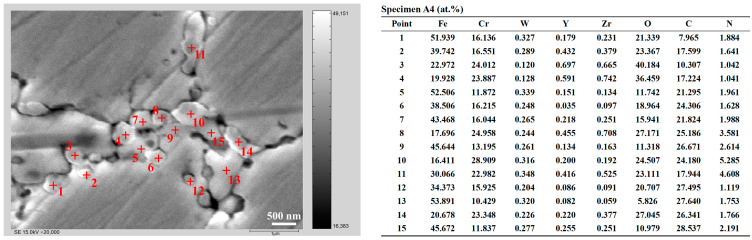
EPMA results of specific compositions of some typical precipitates in specimen A4.

Figure 15 shows the specific compositions of some typical precipitates, distributed at grain boundaries and triple junctions, in specimen A4, which were measured with EPMA. The results of the typical particles show that the precipitates mainly consisted of M_23_C_6_, M_7_C_3_, Cr_2_O_3_, M_2_(C,N), WC, etc. The state of Y_4_Zr_3_O_12_ particles distributed at grain boundaries was not clear, they could be present alone at grain boundaries or dissolved in large precipitates. Overall, the point scan results shown in Figure 15 are consistent with the element distribution results displayed in Figure 14.

**Figure 16:**
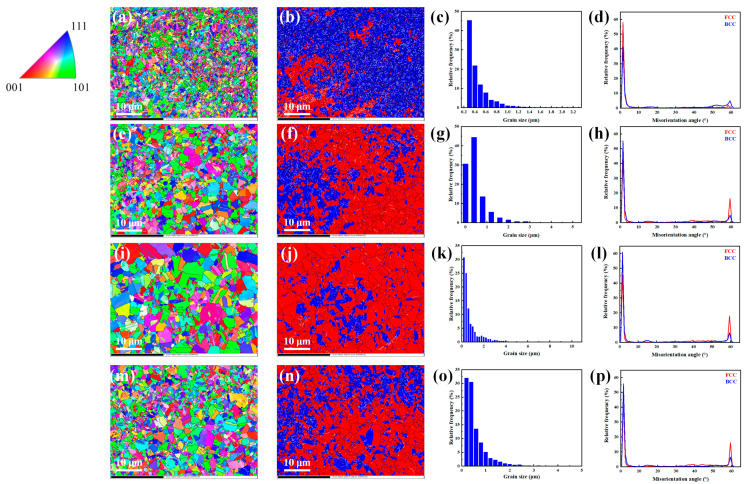
IPF, phase maps (red areas—FCC, blue areas—BCC), grain size, and misorientation angle of the specimens, respectively: (**a**–**d**) A1, (**e**–**h**) A2, (**i**–**l**) A3, and (**m**–**p**) A4.

Figure 16 displays the EBSD analysis results of specimens A1–A4, with noise reduction. The unresolved areas were reasonably eliminated, which might include holes, precipitates, or ultrafine grains affected by high lattice distortion. The red areas correspond to austenite, while the blue areas correspond to ferrite. The white lines represent low angle grain boundaries (LAGB, 2–15°), and the black lines represent high angle grain boundaries (HAGB, > 15°). Similar bimodal grain size distributions have been observed for other ODS alloys prepared by the SPS method [72,73,74,75]. The coarse grains helped to improve the plasticity of the steel, while the fine grains were beneficial to the increase in strength. The orientation imaging maps show that the grain orientations of specimens A1–A4 were essentially random, which is considered to be a typical feature of alloys sintered by SPS [75,76]. The average grain sizes of the specimens were 0.48 μm, 0.65 μm, 0.82 μm and 0.64 μm, respectively, which were obtained by counting more than 3000, 2000, 1400, and 2000 grains, respectively. The average sizes of austenitic grains of the specimens were 0.54 μm, 0.73 μm, 0.96 μm, and 0.72 μm, respectively, while the average sizes of ferritic grains were 0.47 μm, 0.54 μm, 0.59 μm, and 0.57 μm, respectively. Combined with the α/γ grain sizes, the inverse pole figure (IPF) maps, and the phase distribution maps for observation, it can be concluded that most of the ferritic grains were smaller than the austenitic grains. With the increase in sintering temperature, the grains grew and thus the grain size increased. The proportion of LAGB for all specimens was much higher than that of HAGB. Specimen A3 showed the highest proportion of LAGB, which was beneficial for the mechanical properties of the steel.

**Figure 17:**
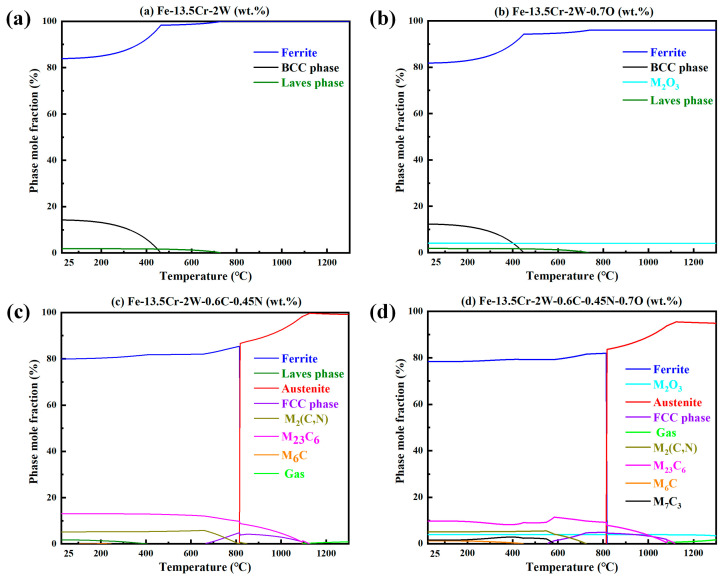
Equilibrium phase compositions of steels with different compositions at different temperatures, calculated with Thermo Calc software: (**a**) Fe–13.5Cr–2W, (**b**) Fe–13.5Cr–2W–0.7O, (**c**) Fe–13.5Cr–2W–0.6C–0.45N, and (**d**) Fe–13.5Cr–2W–0.6C–0.45N–0.7O.

The simulated equilibrium phase compositions of the steels with different compositions, at different temperatures, calculated with the Thermo Calc software, are shown in Figure 17. The following conclusions can be drawn: (i) the matrix of Fe–13.5Cr–2W was fully ferritic at any temperature, under the ideal conditions; (ii) the presence of O would not affect the phase transformation of the matrix at high temperature, but would result in the formation of oxides; (iii) C and N would lead to α→γ transformation at high temperature; (iv) C, N, and O would lead to the formation of M_23_C_6_, Cr_2_O_3_, M_7_C_3_, M_2_(C,N), and other precipitates. It should be noted, that the simulation results were in equilibrium, but the actual preparation process of the steels was a non-equilibrium transformation. A great deal of austenite was not able to transform back to ferrite, due to the rapid cooling rate and the γ-Fe stabilization effect of W. Therefore, the simulation results were approximately consistent with the experimental results, for the phase structure at RT. Additionally, as for the composition of precipitates, the simulation results were also generally consistent with the experimental observations.

### 3.2. Mechanical Properties

The average Vickers microhardness of specimens A1–A4 is shown in Table 6. The high hardness was mainly due to the presence of C and N. Specimen A1 had the highest average hardness, of 598 HV, which was attributed to the poor forming effect caused by the low sintering temperatures. Specifically, the uneven element distribution, lots of dislocations, and high internal stress, would contribute to the increase in hardness, due to the poor forming effect. In addition, the grains of specimen A1 were smaller, so the effect of fine-grain strengthening also led to this result. Comparing the results of specimens A1, A2, and A4, it can be seen that the hardness decreased with the increase in sintering temperature, while the standard deviation decreased as well. This was due to the better forming effect, with a more uniform element distribution and a reduction in dislocations and internal stress. Moreover, the decrease in hardness was also attributed to the increase in grain size. However, the hardness of specimen A3 was higher than that of specimen A2, which was because the Cr-rich phase almost disappeared in specimen A3, and the microstructure of local areas was uniform. However, the local melting of sample A3 led to large differences in the forming effect between regions, thus the hardness fluctuated significantly. In general, there was a certain correlation between the average hardness and the corresponding fluctuation trend, and specimen A4 showed a better comprehensive performance in hardness.

**Figure 18:**
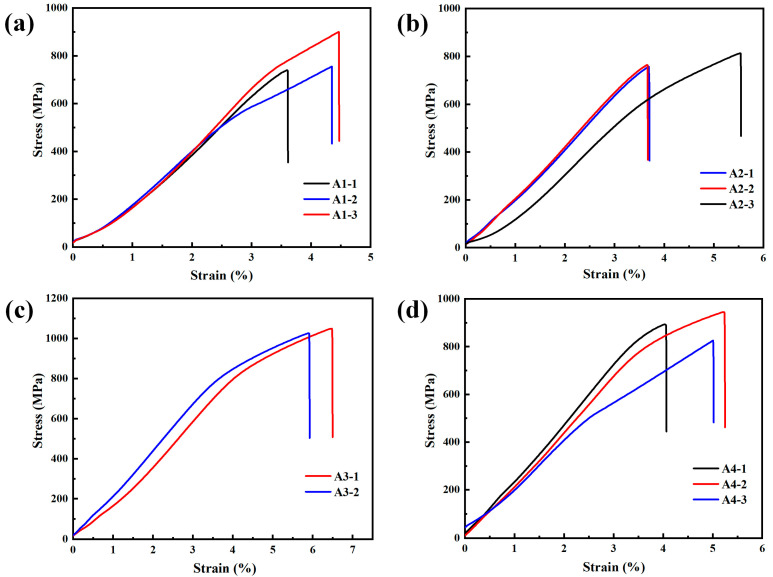
Stress–strain curves of the specimens: (**a**) A1, (**b**) A2, (**c**) A3, and (**d**) A4.

Table 6 and Figure 18 show the ultimate tensile strength (UTS), uniform elongation (UE), and stress–strain curves of specimens A1–A4. It should be noted that as the original sample A3 retained by local melting was very small, only two tensile specimens were used to test the tensile properties of sample A3. For the other samples, three tensile specimens were used to evaluate the tensile properties of each sample. The UTS of specimen A1 was higher than that of specimen A2, which was due to the poor forming effect of specimen A1. The element enrichment in the matrix (such as a Cr-rich phase) and the stress field of a large number of defects at the grain boundaries, made it more difficult for crystal planes to slip, so the UTS of specimen A1 was higher. In addition, the grains of specimen A1 were smaller, and the fine-grain-strengthening effect was also conducive to the increase in strength. The UTSs of specimens A3 and A4 were higher, because the forming effects of the two specimens were significantly improved, and the densities were also higher. Additionally, the main alloying elements and the precipitates were more evenly distributed, and the internal stress was released. Although the larger grain size weakened the fine-grain-strengthening effect, this effect was compensated for by the others. With the increase in temperature at platform 1 or platform 2, the UE would increase as well, and specimen A3 showed a relatively better plasticity, due to the higher sintering temperatures. It is worth pointing out that the dispersed Y_4_Zr_3_O_12_ particles would increase the strength of the specimens, by impeding dislocation movement, which could be explained by the Orowan mechanism [77,78]. The characterization of Y_4_Zr_3_O_12_ nanoparticles should be carefully studied in the near future. From the stress–strain curves, it can be seen that there was no yielding stage in the tensile process of all specimens, which suggests that brittle fracture occurred. The curves were relatively gentle at the last stage, indicating that a slight work-hardening effect occurred. Wu et al. [39] showed that the UTS of Fe–15Cr–2W–0.35Ti–0.6Y_4_Zr_3_O_12_ ODS steel, produced by HIP, was 1006 MPa, with a UE of 15.5% at RT. Li et al. [79] indicated that the UTS of Fe–14Cr–1.5W–0.9Zr–0.45Y_2_O_3_ ODS steel, prepared by HIP, was 981 MPa, with a UE of 16.7% at RT. Li et al. [80] reported that the UTS of Fe–14Cr–2W–0.3Ti–0.3Y_2_O_3_ ODS steel, fabricated by SPS, was ~940 MPa to ~1500 MPa at RT, with sintering temperatures ranging from 950 °C to 1025 °C, and it should be noted that the UTS decreased with the increase in the sintering temperature. Macía et al. [75] prepared Fe–14Cr–3W–5Al–0.4Ti–0.6Zr–0.25Y_2_O_3_ ODS steel, with the same heating rate as that in this study, using the SPS process, and the UTS of the ODS steel was 976 MPa at RT. Compared with the above ODS steels, with comparable compositions, specimen A3 in this study showed a similar UTS, of 1038 MPa, and a lower UE, of 6.2%. The lower UE of specimen A3 was due to the poor forming effect (locally melted), the sub-micron precipitates generated from C, N, and O, and the presence of dissolved C and N in the matrix. However, the Fe–13.5Cr–2W–0.6Y_4_Zr_3_O_12_ ODS steel in this study, still showed a competitive potential in the comprehensive tensile properties, which could be improved by optimizing the SPS process and using purer raw powders.

**Figure 19:**
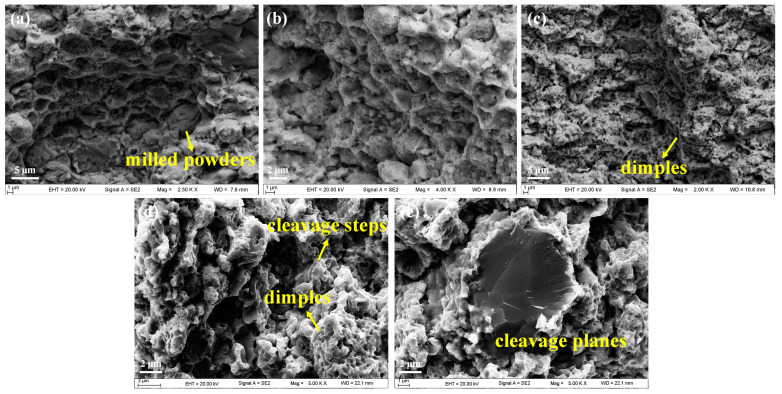
SEM images of the fracture morphology for specimens: (**a**) A1, (**b**) A2, (**c**) A3, (**d**,**e**) A4.

The fracture morphology is shown in Figure 19. An obvious fracture characteristic along grain boundaries could be seen in specimens A1 and A2. As the sintered samples have not been subjected to any interface control treatment, a high density of hard and brittle precipitates were continuously distributed at grain boundaries. These precipitates acted as the stress concentration zones and crack initiation zones, which deteriorated the plasticity of the specimens. There were a few original milled particles remaining in the specimens after sintering, which demonstrated the poor forming effect for specimen A1. It can be concluded that it was intergranular fracture that occurred in specimens A1 and A2. A large number of small and shallow dimples were distributed in specimen A3, indicating the improvement of the plasticity, which was consistent with the results from the stress–strain curves. In addition, some cleavage planes could be observed as well. As for specimen A4, there were widespread regional cleavage steps and several cleavage planes, with unobvious river-like patterns. A few local small dimples and torn edges were also observed. It could be determined that a quasi-cleavage fracture happened in specimen A4. The tensile properties were severely affected by the high-density large precipitates. A feasible solution was to employ a suitable heat treatment process, to facilitate the dissolution of precipitates in the matrix, thus improving the tensile properties of the steels. In general, the effective control of C and N would be a potential route to improving the microstructure and mechanical properties of steels.

## 4. Conclusions

The Y_4_Zr_3_O_12_-added Fe–13.5Cr–2W ODS steels, containing high contents of C and N, were prepared by MA and two-step SPS. The influence of ball-milling time on the microstructure of the alloyed powders and the influence of sintering conditions on the microstructure and mechanical properties of ODS steels, were investigated in detail. The main conclusions can be summarized as follows:

(1) Y_4_Zr_3_O_12_ powders with a grain size of only 3.5 nm, were well prepared by the sol-gel method. During the preparation process of Y_4_Zr_3_O_12_, NH_3_·H_2_O was added, in order to bind Y^3+^ and Zr^4+^ after mixing Y(NO_3_)_3_·6H_2_O and Zr(NO_3_)_4_·5H_2_O in the molten stearic acid. Y_4_Zr_3_O_12_ was stable, due to its high binding energy, during the MA and SPS processes.
(2) MA was an effective process to prepare alloyed powders. After 48 h of ball milling at 300 rpm, a homogeneous element distribution was obtained on the surface of the as-milled powders.
(3) The presence of C and N led to the α-γ dual-phases in the steels at RT, while the fast-sintering characteristic of SPS, and the inhibitory effect of W on C diffusion, also contributed to this result.
(4) The ODS steels displayed a bimodal microstructure, with fine and coarse grains. The Y_4_Zr_3_O_12_ particles were continuously distributed at the grain boundaries and dispersed within the matrix. Some sub-micron precipitates such as M_23_C_6_, Cr_2_O_3_, and M_2_(C,N) were distributed at the grain boundaries and especially at triple junctions. The two temperature platforms of the two-step SPS process, together affected the microstructure and mechanical properties of the steels. When the sintering temperature at platform 1 or platform 2 was increased, the microstructure tended to be more homogeneous. The UTS of specimen A3, sintered with the temperature combination of 750 °C and 1150 °C, reached 1038 MPa, which is similar to the results of other ODS steels with comparable compositions. The designed ODS steel in this study, shows the competitive potential in comprehensive tensile properties, which could be improved by optimizing the SPS process and using purer raw powders.

## Figures and Tables

**Table 1:** Chemical compositions of the alloyed powders (wt.%) and powder sizes (μm).

	Fe	Cr	W	Y–Zr–O			C	N	Ni	Mn
				Y	Zr	O				
Nominal composition	Bal.	13.50	2.00	0.26	0.20	0.14	/			
Tested composition	Bal.	13.46	1.77	0.14	0.18	0.73	0.61	0.45	0.052	0.0051
Powder size	5	1	0.05	/			/			

**Table 2:** Sintering conditions and relative densities of the specimens.

Specimen No.	Temperature Platform 1 (°C)	Dwelling Time 1 (min)	Temperature Platform 2 (°C)	Dwelling Time 2 (min)	Maximum Pressure (MPa)	Relative Density (%)
A1	750	7	1050	7	50	97.1
A2	750		1100			99.1
A3	750		1150			99.3 (locally melted)
A4	800		1100			99.7
A5	800		1150			Melted

**Table 3:** Contents of C and N in the raw powders and as-sintered steel (wt.%).

	C	N
Fe powders	0.70	0.52
Cr powders	0.016	0.0071
W powders	0.019	0.054
As-produced Y–Zr–O powders	1.12	0.11
As-sintered steel	0.61	0.45

**Table 4:** Contents of C and N in the two kinds of Fe powders (wt.%).

	C	N
Atomized Fe powders	0.70	0.52
Electrolytic Fe powders	0.66	0.60

**Table 5:** Different cooling rates and phase structures of specimens at RT.

Specimen No.	Heating Rate (°C/min)	Maximum Temperature (°C)	Holding Time (min)	Cooling Condition	Phase Structure at RT
H1 (as-sintered specimen)	/	/	/	With SPS program	γ + α
H2	10	1100	30	Furnace cooling (<10 °C/min)	α
H3				Air cooling	γ + α
H4				Water cooling	γ + α

**Table 6:** Microhardness, ultimate tensile strength (UTS), and uniform elongation (UE) of the specimens.

Specimen No.	Temperature Platform 1 (°C)	Temperature Platform 2 (°C)	Hardness (HV)	UTS (MPa)	UE (%)
A1	750	1050	598 ± 35	799 ± 72	4.1 ± 0.4
A2	750	1100	522 ± 24	779 ± 25	4.3 ± 0.9
A3	750	1150	543 ± 44	1038 ± 12	6.2 ± 0.3
A4	800	1100	480 ± 16	888 ± 49	4.8 ± 0.5

## Data Availability

The raw/processed date of this study are available from the corresponding author upon reasonable request.

## References

[1] Odette G.R., Alinger M.J., Wirth B.D. (2008). Recent developments in irradiation-resistant steels. Annu. Rev. Mater. Res..

[2] Oksiuta Z., Olier P., de Carlan Y., Baluc N. (2009). Development and characterisation of a new ODS ferritic steel for fusion reactor application. J. Nucl. Mater..

[3] Zhao Q., Yu L.M., Liu Y.C., Huang Y., Ma Z.Q., Li H.J., Wu J.F. (2017). Microstructure and tensile properties of a 14Cr ODS ferritic steel. Mater. Sci. Eng. A.

[4] Toloczko M.B., Gelles D.S., Garner F.A., Kurtz R.J., Abe K. (2004). Irradiation creep and swelling from 400 to 600 °C of the oxide dispersion strengthened ferritic alloy MA957. J. Nucl. Mater..

[5] Kaito T., Narita T., Ukai S., Matsuda Y. (2004). High temperature oxidation behavior of ODS steels. J. Nucl. Mater..

[6] Hu H.L., Zhou Z.J., Liao L., Zhang L.F., Wang M., Li S.F., Ge C.C. (2013). Corrosion behavior of a 14Cr-ODS steel in supercritical water. J. Nucl. Mater..

[7] Kondo K., Aoki S., Yamashita S., Ukai S., Sakamoto K., Hirai M., Kimura A. (2018). Ion irradiation effects on FeCrAl-ODS ferritic steel. Nucl. Mater. Energy.

[8] Yano Y., Ogawa R., Yamashita S., Ohtsuka S., Kaito T., Akasaka N., Inoue M., Yoshitake T., Tanaka K. (2011). Effects of neutron irradiation on tensile properties of oxide dispersion strengthened (ODS) steel claddings. J. Nucl. Mater..

[9] Kimura A., Kasada R., Iwata N., Kishimoto H., Zhang C.H., Isselin J., Dou P., Lee J.H., Muthukumar N., Okuda T. (2011). Development of Al added high-Cr ODS steels for fuel cladding of next generation nuclear systems. J. Nucl. Mater..

[10] Kimura A., Kayano H., Misawa T., Matsui H. (1994). Designation of alloy composition of reduced-activation martensitic steel. J. Nucl. Mater..

[11] Ukai S., Ohtsuka S., Kaito T., de Carlan Y., Ribis J., Malaplate J., Pascal Y. (2017). Oxide dispersion-strengthened/ferrite-martensite steels as core materials for Generation IV nuclear reactors. Structural Materials for Generation IV Nuclear Reactors.

[12] Mukhopadhyay D.K., Froes F.H., Gelles D.S. (1998). Development of oxide dispersion strengthened ferritic steels for fusion. J. Nucl. Mater..

[13] Olier P., Bougault A., Alamo A., de Carlan Y. (2009). Effects of the forming processes and Y_2_O_3_ content on ODS-Eurofer mechanical properties. J. Nucl. Mater..

[14] de Castro V., Leguey T., Munoz A., Monge M.A., Pareja R., Marquis E.A., Lozano-Perez S., Jenkins M.L. (2009). Microstructural characterization of Y_2_O_3_ ODS-Fe-Cr model alloys. J. Nucl. Mater..

[15] Gwalani B., Pohan R.M., Waseem O.A., Alam T., Hong S.H., Ryu H.J., Banerjee R. (2019). Strengthening of Al_0.3_CoCrFeMnNi-based ODS high entropy alloys with incremental changes in the concentration of Y_2_O_3_. Scr. Mater..

[16] Sun D.J., Liang C.Y., Shang J.L., Yin J.H., Song Y.R., Li W.Z., Liang T.Q., Zhang X.H. (2016). Effect of Y_2_O_3_ contents on oxidation resistance at 1150 °C and mechanical properties at room temperature of ODS Ni-20Cr-5Al alloy. Appl. Surf. Sci..

[17] Massey C.R., Hoelzer D.T., Unocic K.A., Osetskiy Y.N., Edmondson P.D., Gault B., Zinkle S.J., Terrani K.A. (2020). Extensive nanoprecipitate morphology transformation in a nanostructured ferritic alloy due to extreme thermomechanical processing. Acta Mater..

[18] Zhuang Y., Zhang X., Peng T., Fan H., Zhang X., Yan Q., Volinsky A.A. (2020). Effects of yttrium oxides on the microstructure and mechanical properties of 15-15Ti ODS alloy fabricated by casting. Mater. Charact..

[19] Wu Z.F., Xu L.D., Chen H.Q., Liang Y.X., Du J.L., Wang Y.F., Zhang S.L., Cai X.C., Sun B.R., Zhang J. (2022). Significant suppression of void swelling and irradiation hardening in a nanograined/nanoprecipitated 14YWT-ODS steel irradiated by helium ions. J. Nucl. Mater..

[20] Ren J., Yu L.M., Liu Y.C., Liu C.X., Li H.J., Wu J.F. (2018). Effects of Zr addition on strengthening mechanisms of Al-alloyed high-Cr ODS steels. Materials.

[21] Zhao Q., Yu L.M., Ma Z.Q., Li H.J., Wang Z.M., Liu Y.C. (2018). Hot deformation behavior and microstructure evolution of 14Cr ODS steel. Materials.

[22] Song L.L., Yang X.Y., Zhao Y.Y., Wang W., Mao X.D. (2019). Si-containing 9Cr ODS steel designed for high temperature application in lead-cooled fast reactor. J. Nucl. Mater..

[23] Xu Z.Y., Song L.L., Zhao Y.Y., Liu S.J. (2021). The formation mechanism and effect of amorphous SiO_2_ on the corrosion behaviour of Fe-Cr-Si ODS alloy in LBE at 550 °C. Corros. Sci..

[24] Wang Y., Zhou Z.J., Jia H.D., Gao R., Ran M.R., Zheng W.Y., Zhang M.C., Li H., Zhang J.Q., Zeng X.Q. (2023). Understanding the excellent corrosion resistance of Fe-12Cr ODS alloys with and without Si in supercritical CO_2_ through advanced characterization. Corros. Sci..

[25] Husak R., Hadraba H., Chlup Z., Heczko M., Kruml T., Puchy V. (2019). ODS Eurofer steel strengthened by Y-(Ce, Hf, La, Sc, and Zr) complex oxides. Metals.

[26] Yan P.Y., Yu L.M., Liu Y.C., Liu C.X., Huijun L.J., Wu J.F. (2018). Effects of Hf addition on the thermal stability of 16Cr-ODS steels at elevated aging temperatures. J. Alloys Compd..

[27] He S.Y., Qian Q., Huang Z., Gong Y.X., Chen J.J., Wang Y.R., Jiang Y. (2021). Nucleation of Y-Si-O nano-clusters in multi-microalloyed nano-structured ferritic alloys: A first-principles study. Acta Metall. Sin.-Engl. Lett..

[28] Jiang Y., Smith J.R., Odette G.R. (2009). Formation of Y-Ti-O nanoclusters in nanostructured ferritic alloys: A first-principles study. Phys. Rev. B.

[29] Murali D., Panigrahi B.K., Valsakumar M.C., Chandra S., Sundar C.S., Raj B. (2010). The role of minor alloying elements on the stability and dispersion of yttria nanoclusters in nanostructured ferritic alloys: An ab initio study. J. Nucl. Mater..

[30] Dou P., Kimura A., Kasada R., Okuda T., Inoue M., Ukai S., Ohnuki S., Fujisawa T., Abe F. (2014). TEM and HRTEM study of oxide particles in an Al-alloyed high-Cr oxide dispersion strengthened steel with Zr addition. J. Nucl. Mater..

[31] Dou P., Qiu L.L., Jiang S.M., Kimura A. (2019). Crystal and metal/oxide interface structures of nanoparticles in Fe-16Cr-0.1Ti-0.35Y_2_O_3_ ODS steel. J. Nucl. Mater..

[32] Jiang Y., Smith J.R., Odette G.R. (2010). Prediction of structural, electronic and elastic properties of Y_2_Ti_2_O_7_ and Y_2_TiO_5_. Acta Mater..

[33] Hirata A., Fujita T., Wen Y.R., Schneibel J.H., Liu C.T., Chen M.W. (2011). Atomic structure of nanoclusters in oxide-dispersion-strengthened steels. Nat. Mater..

[34] Hin C., Wirth B.D. (2010). Formation of Y_2_O_3_ nanoclusters in nano-structured ferritic alloys: Modeling of precipitation kinetics and yield strength. J. Nucl. Mater..

[35] Pazos D., Suarez M., Fernandez A., Fernandez P., Iturriza I., Ordas N. (2019). Microstructural comparison of Oxide Dispersion Strengthened Fe-14Cr steels produced by HIP and SPS. Fusion Eng. Des..

[36] Zhang L.Y., Yu L.M., Liu Y.C., Liu C.X., Li H.J., Wu J.F. (2017). Influence of Zr addition on the microstructures and mechanical properties of 14Cr ODS steels. Mater. Sci. Eng. A.

[37] Simondon E., Giroux P.F., Chaffron L., Fitch A., Castany P., Gloriant T. (2018). Mechanical synthesis of nanostructured Y_2_Ti_2_O_7_ pyrochlore oxides. Solid State Sci..

[38] Liu T., Wang L.B., Wang C.X., Shen H.L., Zhang H.T. (2015). Feasibility of using Y_2_Ti_2_O_7_ nanoparticles to fabricate high strength oxide dispersion strengthened Fe-Cr-Al steels. Mater. Des..

[39] Wu Y., Zhao H.Z., Li J.K., Zhang Y.Y., Liu T. (2021). Effects of Y_4_Zr_3_O_12_ addition on the microstructure and mechanical properties of Fe-15Cr-2W-0.35Ti ODS steels. Mater. Sci. Eng. A.

[40] Sun Q.X., Fang Q.F., Zhou Y., Xia Y.P., Zhang T., Wang X.P., Liu C.S. (2013). Development of oxide dispersion strengthened ferritic steel prepared by chemical reduction and mechanical milling. J. Nucl. Mater..

[41] Mazumder B., Parish C.M., Bei H., Miller M.K. (2015). The role of processing route on the microstructure of 14YWT nanostructured ferritic alloy. J. Nucl. Mater..

[42] Suryanarayana C. (2001). Mechanical alloying and milling. Prog. Mater. Sci..

[43] Auger M.A., de Castro V., Leguey T., Munoz A., Pareja R. (2013). Microstructure and mechanical behavior of ODS and non-ODS Fe-14Cr model alloys produced by spark plasma sintering. J. Nucl. Mater..

[44] Guillon O., Gonzalez-Julian J., Dargatz B., Kessel T., Schierning G., Rathel J., Herrmann M. (2014). Field-assisted sintering technology/spark plasma sintering: Mechanisms, materials, and technology developments. Adv. Eng. Mater..

[45] Matizamhuka W.R. (2016). Spark plasma sintering (SPS)-An advanced sintering technique for structural nanocomposite materials. J. South. Afr. Inst. Min. Metall..

[46] Munir Z.A., Anselmi-Tamburini U., Ohyanagi M. (2006). The effect of electric field and pressure on the synthesis and consolidation of materials: A review of the spark plasma sintering method. J. Mater. Sci..

[47] Fu J., Brouwer J.C., Richardson I.M., Hermans M.J.M. (2019). Effect of mechanical alloying and spark plasma sintering on the microstructure and mechanical properties of ODS Eurofer. Mater. Des..

[48] Peng S.B., Lu Z., Li X.L., Yu L. (2022). A comparative study of microstructure and mechanical properties of ODS CrFeNi-based medium- and high-entropy alloys. J. Alloys Compd..

[49] Frelek-Kozak M., Kurpaska L., Wyszkowska E., Jagielski J., Jozwik I., Chmielewski M. (2018). Evaluation of consolidation method on mechanical and structural properties of ODS RAF steel. Appl. Surf. Sci..

[50] Shi W.Z., Yu L.M., Liu C.X., Ma Z.Q., Li H.J., Wang Z.M., Liu Y.C., Gao Q.Z., Wang H. (2022). Evolution of Y_2_O_3_ precipitates in ODS-316 L steel during reactive-inspired ball-milling and spark plasma sintering processes. Powder Technol..

[51] Lóh N.J., Simão L., Faller C.A., De Noni A., Montedo O.R.K. (2016). A review of two-step sintering for ceramics. Ceram. Int..

[52] Mihalache V., Mercioniu I., Velea A., Palade P. (2019). Effect of the process control agent in the ball-milled powders and SPS-consolidation temperature on the grain refinement, density and Vickers hardness of Fe14Cr ODS ferritic alloys. Powder Technol..

[53] Diouf S., Molinari A. (2012). Densification mechanisms in spark plasma sintering: Effect of particle size and pressure. Powder Technol..

[54] Oksiuta Z., Baluc N. (2009). Optimization of the chemical composition and manufacturing route for ODS RAF steels for fusion reactor application. Nucl. Fusion.

[55] Song L.L., Liu S.J., Mao X.D. (2016). Microstructure evolution of the oxide dispersion strengthened CLAM steel during mechanical alloying process. Fusion Eng. Des..

[56] Macía E., Garcia-Junceda A., Serrano M., Hong S.J., Campos M. (2021). Effect of mechanical alloying on the microstructural evolution of a ferritic ODS steel with (Y-Ti-Al-Zr) addition processed by Spark Plasma Sintering (SPS). Nucl. Eng. Technol..

[57] Garcia-Cabezon C., Blanco Y., Rodriguez-Mendez M.L., Martin-Pedrosa F. (2017). Characterization of porous nickel-free austenitic stainless steel prepared by mechanical alloying. J. Alloys Compd..

[58] Suehiro M., Liu Z.K., Agren J. (1996). Effect of niobium on massive transformation in ultra low carbon steels: A solute drag treatment. Acta Mater..

[59] Fossaert C., Rees G., Maurickx T., Bhadeshia H. (1995). The effect of niobium on the hardenability of microalloyed austenite. Metall. Mater. Trans. A.

[60] Simielli E.A., Yue S., Jonas J.J. (1992). Recrystallization kinetics of microalloyed steels deformed in the intercritical region. Metall. Trans. A.

[61] Bradley J.R., Aaronson H.I. (1981). Growth kinetics of grain boundary ferrite allotriomorphs in Fe-C-X alloys. Metall. Trans. A.

[62] Purdy G.R., Brechet Y.J.M. (1995). A solute drag treatment of the effects of alloying elements on the rate of the proeutectoid ferrite transformation in steels. Acta Metall. Mater..

[63] Durand A., Sornin D., Tache O., Guilbert T., Brisset F., Delbes L., Baptiste B., Baudin T., Loge R. (2023). Stability of untransformed ferrite in 10Cr ODS steel. J. Nucl. Mater..

[64] Durand A., Sornin D., Carlan Y.d., Spartacus G., Brisset F., Delbes L., Baptiste B., Baudin T., Logé R. (2021). Characterization of untransformed ferrite in 10Cr and 12Cr ODS steels. Materialia.

[65] Tokita M. (1999). Development of large-size ceramic/metal bulk FGM fabricated by spark plasma sintering. Mater. Sci. Forum.

[66] Sulima I., Putyra P., Hyjek P., Tokarski T. (2015). Effect of SPS parameters on densification and properties of steel matrix composites. Adv. Powder Technol..

[67] Gottstein G., Shvindlerman L.S., Zhao B. (2010). Thermodynamics and kinetics of grain boundary triple junctions in metals: Recent developments. Scr. Mater..

[68] Chen C.R., Li S.X., Wen J.L., Jia W.P. (2000). Finite element analysis about effects of stiffness distribution on stresses and elastic strain energy near the triple junction in a tricrystal. Mater. Sci. Eng. A.

[69] Miura H., Andiarwanto S., Sato K., Sakai T. (2002). Preferential dynamic nucleation at triple junction in copper tricrystal during high-temperature deformation. Mater. Trans..

[70] Hallberg H., Ristinmaa M. (2013). Microstructure evolution influenced by dislocation density gradients modeled in a reaction-diffusion system. Comput. Mater. Sci..

[71] Yoo M.H., Trinkaus H. (1983). Crack and cavity nucleation at interfaces during creep. Metall. Trans. A.

[72] Allahar K.N., Burns J., Jaques B., Wu Y.Q., Charit I., Cole J., Butt D.P. (2013). Ferritic oxide dispersion strengthened alloys by spark plasma sintering. J. Nucl. Mater..

[73] Meza A., Macía E., Chekhonin P., Altstadt E., Rabanal M.E., Torralba J.M., Campos M. (2022). The effect of composition and microstructure on the creep behaviour of 14 Cr ODS steels consolidated by SPS. Mater. Sci. Eng. A.

[74] Ninawe P.S., Ganesh S., Sai Karthik P., Chandrasekhar S.B., Vijay R. (2022). Microstructure and mechanical properties of spark plasma sintered austenitic ODS steel. Adv. Powder Technol..

[75] Macía E., García-Junceda A., Serrano M., Hernández-Mayoral M., Diaz L.A., Campos M. (2019). Effect of the heating rate on the microstructure of a ferritic ODS steel with four oxide formers (Y-Ti-Al-Zr) consolidated by spark plasma sintering (SPS). J. Nucl. Mater..

[76] Garcia-Junceda A., Macia E., Garbiec D., Serrano M., Torralba J.M., Campos M. (2020). Effect of small variations in Zr content on the microstructure and properties of ferritic ODS steels consolidated by SPS. Metals.

[77] Li W., Xu H., Sha X., Meng J., Wang W., Kang C., Zhang X., Wang Z. (2018). Microstructural characterization and strengthening mechanisms of a 15Cr-ODS steel produced by mechanical alloying and Spark Plasma Sintering. Fusion Eng. Des..

[78] Steckmeyer A., Praud M., Fournier B., Malaplate J., Garnier J., Béchade J.L., Tournié I., Tancray A., Bougault A., Bonnaillie P. (2010). Tensile properties and deformation mechanisms of a 14Cr ODS ferritic steel. J. Nucl. Mater..

[79] Li Y., Zhang L., Long D., Yu L., Li H. (2021). The Precipitated Particle Refinement in High-Cr ODS Steels by Microalloying Element Addition. Materials.

[80] Li Z., Lu Z., Xie R., Lu C., Liu C. (2016). Effect of spark plasma sintering temperature on microstructure and mechanical properties of 14Cr-ODS ferritic steels. Mater. Sci. Eng. A.

